# Whole-Genome Sequence of *Ralstonia solanacearum* P673, a Strain Capable of Infecting Tomato Plants at Low Temperatures

**DOI:** 10.1128/genomeA.00106-14

**Published:** 2014-02-20

**Authors:** Ana M. Bocsanczy, Jose C. Huguet-Tapia, David J. Norman

**Affiliations:** a Department of Plant Pathology, University of Florida, IFAS, Mid-Florida Research and Education Center, Apopka, Florida, USA; b Department of Plant Pathology, University of Florida, Gainesville, Florida, USA

## Abstract

*Ralstonia solanacearum* is the causal agent of bacterial wilt, one of the most destructive bacterial plant diseases. We present the whole-genome sequence of the strain P673 (phylotype IIB, sequevar 4). This strain is capable of producing disease in tomato plants at low temperatures. P673 has 311 unique genes.

## GENOME ANNOUNCEMENT

*Ralstonia solanacearum* (Smith 1896) Yabuuchi et al. 1996 is a Gram-negative soil-borne bacterium that causes bacterial wilt, one of the most destructive bacterial diseases in tropical, subtropical, and recently some temperate areas of the world (1, 2). *R. solanacearum* exhibits such phenotypic and genotypic diversity that it is considered a species complex (3). All established populations of *R. solanacearum* found in the southern United States have been classified as race 1 and biovars 1 and 3 (4). P673 is a race 1 biovar 1 (R1B1) strain with special characteristics. It was isolated from an *Epipremnum aureum* (pothos) ornamental plant (5) and is able to infect tomato and, to a lesser degree, potato plants at 18°C in chamber tests, when inoculated naturally through the soil (6). It was classified as phylotype IIb, sequevar 4, which places it in the same group as the exotic strains in the Caribbean that have been detected in pond and irrigation water in Florida (7).

Genomic DNA was extracted using the UltraClean microbial DNA isolation kit (Mo Bio Laboratories, Inc.), according to the manufacturer’s instructions. A combination of two Illumina single-read and two 454 shotgun libraries produced >18 million reads and an additional 152,375 reads, respectively. The reads were assembled into 251 contigs using the hybrid strategy MIRA software version 3.05 (8). Contigs were annotated by the Prokaryotic Genomes Annotation Pipeline (PGAP) at NCBI (9). There are 4,690 coding sequences (CDSs), 2 rRNAs, 3 noncoding RNAs (ncRNAs), and 45 tRNAs predicted. The annotated contigs were deposited in the NCBI database. Additionally, contigs were assembled in two replicons or gapped chromosomes by the Geneious 6.0 software (Biomatters Ltd.) using the genomic sequence of the closely related strain *R. solanacearum* Po82 (10) as a reference. The chromosome is represented by 3.48 Mbp and the megaplasmid by 1.91 Mbp. The overall length of approximately 5.39 Mbp is shorter than the sequence of Po82 (5.43 Mbp) (10). The overall G+C content of the P673 genome is 66.9%.

Biological functions were assigned to 63% of the predicted proteins. The most represented categories were amino acid, protein, carbohydrate, and lipid metabolism (30%) and stress response, virulence, and cell wall biogenesis (3% each). Hypothetical protein and no-match categories were assigned to 37% of proteins. A comparison of P673 replicons with those of 7 complete genome sequences (10, 11) revealed that 311 genes are unique to P673. Elucidating the genomic differences between P673 and similar strains that do not infect their host at low temperatures will facilitate the discovery of key cool virulence proteins.

### Nucleotide sequence accession numbers.

This whole-genome shotgun project has been deposited at DDBJ/EMBL/GenBank under the accession number JALO00000000. The version described in this paper is version JALO01000000.
